# Multiple asynchronous recurrence as a predictive factor for refractoriness against locoregional and surgical therapy in patients with intermediate-stage hepatocellular carcinoma

**DOI:** 10.1038/s41598-024-61611-4

**Published:** 2024-05-13

**Authors:** Ryosuke Kasuga, Nobuhito Taniki, Po-Sung Chu, Masashi Tamura, Takaya Tabuchi, Akihiro Yamaguchi, Shigeo Hayatsu, Jun Koizumi, Keisuke Ojiro, Hitomi Hoshi, Fumihiko Kaneko, Rei Morikawa, Fumie Noguchi, Karin Yamataka, Shingo Usui, Hirotoshi Ebinuma, Osamu Itano, Yasushi Hasegawa, Yuta Abe, Minoru Kitago, Masanori Inoue, Seishi Nakatsuka, Masahiro Jinzaki, Yuko Kitagawa, Takanori Kanai, Nobuhiro Nakamoto

**Affiliations:** 1 Division of Gastroenterology and Hepatology, Department of Internal Medicine, Keio University School of Medicine, 35 Shinanomachi, Shinjuku-ku, Tokyo, 160-8582 Japan; 2 Department of Radiology, Keio University School of Medicine, Tokyo, Japan; 3 Division of Gastroenterology, Department of Internal Medicine, National Hospital Organization Saitama National Hospital, Saitama, Japan; 4 Department of Surgery, National Hospital Organization Saitama National Hospital, Saitama, Japan; 5 Department of Diagnostic Radiology and Radiation Oncology, School of Medicine, Chiba University, Chiba, Japan; 6 Department of Gastroenterology, Ichikawa General Hospital, Tokyo Dental College, Chiba, Japan; 7 Department of Gastroenterology and Hepatology, Saitama City Hospital, Saitama, Japan; 8 Department of Gastroenterology, School of Medicine, International University of Health and Welfare, Chiba, Japan; 9 Department of Hepato-Biliary-Pancreatic and Gastrointestinal Surgery, International University of Health and Welfare School of Medicine, Chiba, Japan; 10 Department of Surgery, Keio University School of Medicine, Tokyo, Japan

**Keywords:** Hepatocellular carcinoma, Treatment algorithm, Transarterial chemoembolization (TACE), Ablation, Hepatic resection, Liver cancer, Hepatocellular carcinoma

## Abstract

Development of subclassification of intermediate-stage hepatocellular carcinoma (HCC) by treatment suitability is in demand. We aimed to identify predictors that define treatment refractoriness against locoregional(transarterial chemoembolization(TACE) or thermal ablation) and surgical therapy. This multicenter retrospective study enrolled 1167 HCC patients between 2015 and 2021. Of those, 209 patients were initially diagnosed with intermediate-stage HCC. Treatment refractoriness was defined as clinical settings that meets the following untreatable progressive conditions by TACE (1) 25% increase of intrahepatic tumor, (2) transient deterioration to Child–Pugh class C, (3) macrovascular invasion or extrahepatic spread, within one year. We then analyzed factors contributing to treatment refractoriness. The Child–Pugh score/class, number of tumors, infiltrative radiological type, and recurrence were significant factors. Focusing on recurrence as a predictor, median time to untreatable progression (TTUP) was 17.2 months in the recurrence subgroup whereas 35.5 months in the initial occurrence subgroup (HR, 2.06; 95% CI, 1.44–2.96; P = 0.001). Median TTUP decreased in cases with more later times of recurrence (3–5 recurrences, 17.3 months; ≥ 6 recurrences, 7.7 months). Recurrence, even more at later times, leads to increased treatment refractoriness. Early introduction of multidisciplinary treatment should be considered against HCC patients after multiple recurrent episodes.

## Introduction

Hepatocellular carcinoma (HCC) is the third leading cause of cancer-related death in 46 countries and the top five cause of cancer-related death in 90 countries worldwide^1^. Between 2020 and 2040, the number of liver cancer is predicted to increase by 55.0% with 1.4 million new diagnoses^2^.

The Barcelona Clinic Liver Cancer (BCLC) prognosis and treatment strategy is a widely accepted staging system for HCC that incorporates treatment recommendations and prognosis based on patient and tumor characteristics, and underlying liver function. Treatment selection for HCC has generally been determined according to the BCLC staging system. The BCLC staging system classifies HCC into five stages: very early (0), early (A), intermediate (B), advanced (C), and terminal (D). According to these criteria, BCLC intermediate-stage HCC includes patients with preserved liver function and > 3 lesions of any size or 2–3 lesions > 30 mm in diameter without major vessel involvement or extrahepatic metastasis^3^. Although the BCLC intermediate-stage comprises a highly heterogeneous patient population, treatment selection needs to be determined by individual clinical settings^4,5^.

The BCLC HCC staging system was updated in 2022, and the intermediate-stage was divided into three groups by suitability for treatment options: liver transplant, trans-arterial chemoembolization (TACE), and systemic chemotherapy^2^. Patients who are refractory to TACE can benefit from switching treatment to systemic chemotherapy^6,7^ or a combination therapy of systemic chemotherapy and TACE^8^. This evidence suggests that early introduction of systemic chemotherapy is effective against certain populations with intermediate-stage HCC. Surgical resection or thermal ablation has also been reported to be effective in certain populations with intermediate-stage HCC^9,10^. Thus, TACE is probably overused and may not be appropriate for all patients with intermediate-stage HCC^4^. In addition, patients with solitary large HCC > 5 cm beyond the Milan criteria also include certain population to be unsuitable for surgical resection^11,12^. Early recurrence after curative surgery can occur in some patients. In such cases, HCCs are represented by histologically malignant types or microscopic invasion^13,14^, and patients should be considered for multidisciplinary treatment.

Previous studies have revealed several conditions that contribute to TACE refractoriness. The magnitude of the tumor burden is the most used predictive factor for treatment refractoriness of HCC because of its reliability and feasibility. The up-to-7 criteria, with seven being the sum of the diameter of the largest tumor (in centimeter) and the number of tumors, were initially developed for predicting mortality after liver transplantation in patients with HCC beyond the Milan criteria^15^. This concept has been applied to the subclassification of BCLC stage B HCC for predicting TACE refractoriness, and its feasibility has been advocated^16–18^ and validated in several cohorts^19–25^. Moreover, radiological features are also used to predict the malignant potential of HCC. HCCs with intact tumor capsules are associated with lower recurrence rates^26^ whereas HCCs without intact capsules, which are represented by unencapsulated or infiltrative radiological features, have a worse prognosis^27^. These clinical investigations indicated that infiltrative radiological features have the potential to predict HCC that is intolerant against locoregional and surgical therapy; however, there are critical concerns regarding the accuracy of radiological imaging examinations in determining histological gross classification^28^. Further strategies to predict treatment refractoriness inducible factors, such as unfavorable histological features^29^ or driver events of genomic and transcriptional alterations, are clinically in demand.

In the current study, we aimed to develop a predictive factor of treatment refractoriness to locoregional(including TACE and thermal ablation) and surgical therapy in patients with BCLC intermediate-stage HCC, to facilitate the selection of optimal treatment options without evaluating the histological or genetic background of the tumor or treatment responsiveness.

## Materials and methods

### Study design and patients

This retrospective, multicenter, observational study enrolled 1167 patients who were recruited from four liver centers across Japan (Keio University Hospital, National Hospital Organization Saitama Hospital, Tokyo Dental College Ichikawa General Hospital, and Saitama City Hospital) between January 2015 and March 2021. The inclusion criterion for registration was patients who were initially diagnosed with BCLC intermediate-stage including solitary large (> 5 cm) HCC. The major features of HCC include arterial phase hyperenhancement, observation size, washout appearance, enhancing capsule appearance, and threshold growth on multiphase contrast-enhanced CT or MRI according to the American Association for the Study of Liver Diseases criteria and Liver Imaging Reporting and Data System Version 2018^30,31^. Prior resection, ablation, TACE, or stereotactic radiotherapy was allowed whenever curative therapy was achieved. However, no prior systemic therapy was allowed. Previous curative therapy was defined as achieving a complete response (CR) based on the modified Response Evaluation Criteria in Solid Tumors (mRECIST) within 1–3 months after previous treatment.

The study protocol was approved by the Keio University School of Medicine Research Ethics Committee, approval number [No. 20160227]. The study was conducted in accordance with the principles of the 1975 Declaration of Helsinki and Ethical Guidelines for Medical and Health Research involving Human Subjects (Ministry of Education, Culture, Sports, Science, and Technology and Ministry of Health, Labour and Welfare; Japan). The need for written or oral informed consent was waived by the ethics review committees of the Keio University School of Medicine owing to the retrospective nature of the study and to the difficulty of obtaining consent for reason that many participants had died. In accordance with governmental guidelines, we release information on research projects, and offers opt-out opportunities using the official website that allow research participants to withdraw their consent or forbid researchers to use their data for research.

### Medical care

The locoregional (including TACE and thermal ablation) and surgical therapy was administered by experienced investigators at each institution. The generalized treatment protocol was formulated by a multidisciplinary team. Surgical resection or thermal ablation therapy was selected if all regions were predicted to be completely treatable based on the preoperative evaluation. The ablation procedures were operated under real-time ultrasound guidance using radiofrequency ablation (RFA) with cooled-tip RF system™ (Medtronic) or VIVA RF system™ (STARmed) or microwave ablation (MWA) with a cooled-shaft antenna Emprint ablation system™ (Medtronic). Conventional TACE consisted of intra-arterial injection of lipiodol plus epirubicin or miriplatin, followed by injection of an embolic agent (Gelpart™) to interrupt blood flow. Drug-eluting bead transarterial chemoembolization was performed either selectively or super-selectively with 100–300 μm DC- Beads™ (Eisai) loading epirubicin with 50 mg/2 mL of beads. The procedure protocol for RFA/MWA and TACE was determined by the sites/investigators. Therefore, the main findings were validated in each site by the respective analyses to prevent any imbalance. Patients underwent CT or MRI assessment within 3 months from the initiation of locoregional and surgical therapies, and every 3–6 months thereafter. Imaging assessments were retrospectively reviewed by at least two independent investigators according to the mRESIST for HCC.

### Baseline evaluation

Baseline characteristics were evaluated within 1 month of locoregional and surgical therapy. The data included age, sex, body mass index, underlying liver disease, liver function, Child–Pugh score/class and albumin-bilirubin (ALBI) score/modified albumin-bilirubin (mALBI) grade^32^, serum biochemistry data, alpha-fetoprotein and des-γ-carboxy prothrombin levels, tumor characteristics, number of tumors, largest tumor diameter, radiological tumor appearance, and history of HCC. We subclassified HCCs into two radiological features: nodular or infiltrative, based on contrast-enhanced CT or MRI findings. Nodular types were defined as tumors with a clear round shape, pathologically corresponding to a single nodular type. Infiltrative types were defined as tumors with perinodular growth or tumor clustering with small and confluent nodules, pathologically corresponding to a single nodule with extranodular growth and confluent multinodular type.

### Outcome assessment

Progressive disease was defined as untreatable progression by TACE based on the criteria used in the TACTICS trial^8^: (1) intrahepatic tumor progression (25% increase versus [vs.] baseline); (2) transient deterioration of liver function to Child–Pugh score/class C; and (3) major vessel involvement or extrahepatic spread. Time to untreatable progression by TACE (TTUP) was defined as the time from the date of initiation of locoregional and surgical therapy for the first diagnosis of BCLC intermediate-stage including solitary large HCC to the date of confirming radiological untreatable progression by TACE according to the mRECIST criteria. We divided the patients into responder and refractory groups based on their responsiveness to locoregional and surgical therapies. We defined patients with a TTUP < 1 year as refractory to locoregional and surgical therapies. We also evaluated the time interval for HCC recurrence in these patients. The time interval was defined as the duration between the time of confirmation to achieve mRECIST CR by previous treatment and the time of inclusion in the current study.

### Statistical analysis

Data were analyzed using SPSS version 28 (IBM Corp., Armonk, NY, USA) and are expressed as medians with interquartile ranges or as averages ± standard deviations, as appropriate. Graphs and linear correlations were constructed using Prism 9.0 (GraphPad Software, Inc. San Diego, CA, USA). Differences between the groups were assessed using the Student t-test. Categorical variables were assessed using the chi-square test. Logistic regression analysis was performed to determine the factors associated with treatment refractoriness using a set of covariates that were significant factors in the univariate analyses. Relative risks are expressed as odds ratios (OR) with 95% confidence intervals (95% CIs). Hazard ratios (HRs) and 95% confidence intervals (CIs) were estimated using Cox proportional hazards models. A post hoc analysis indicated that a minimum of 197 patients would be required to achieve a power of at least 80% at a significance level of 0.05 for the Chi-square test between the refractory and responder groups in the case of recurrence or non-recurrence, assuming an effect size of 0.2. Area under the receiver operating characteristic (AUROC) analysis was performed to determine the usefulness of various factors for predicting treatment refractoriness and to generate favorable cut-off values based on the Youden Index. Kaplan–Meier analysis was used to determine the cumulative percentage of untreatable progression by TACE, and differences between the groups were assessed using the log-rank test. Internal validity was estimated using bootstrapping validation. We ran logistic regression analysis with 1000 random replications of our dataset and generated 95% bootstrap CIs for the significant variables extracted by multivariate analysis. For all analyses, a significance level of *P* < 0.05 was considered significant.

## Results

### Patient characteristics

**Figure 1:**
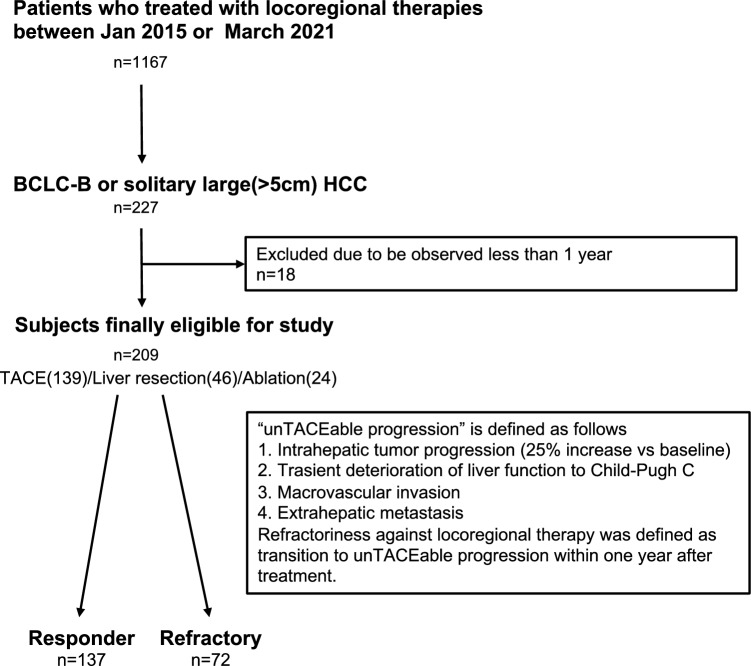
Flow chart of eligible patients.

After registration, 227 patients met the inclusion criteria, and 18 patients were excluded because of an observation period of < 1 year. Finally, 209 patients from four sites were included in the study (Fig. 1). A total of 139 patients (66.5%) underwent TACE, 46 (22.0%) underwent surgical resection, and 24 (11.5%) underwent RFA or MWA. The patients’ background characteristics are summarized in Table 1. The median age at treatment initiation was 74 years, and 75.6% of the patients were male. The underlying liver diseases were hepatitis B virus (12.2%), hepatitis C virus (33.0%), alcoholic hepatitis (19.1%), nonalcoholic steatohepatitis (19.6%), primary biliary cholangitis (1.4%), and cryptogenic (14.8%). Most patients had preserved liver function, as assessed by the Child–Pugh score/class and mALBI grade: 85.6% of patients were classified as Child–Pugh score/class A, and the remaining 14.3% as Child–Pugh score/class B. Among mALBI grades, mALBI grade 1 (44.5%) was the most prominent, followed by mALBI grades 2b (33.0%), 2A (21.5%), and 3 (1.0%). Of the patients, 49.3% had recurrent HCC after curative therapy; previous treatments included surgical resection (25.2%), radiofrequency or microwave ablation (40.8%), and TACE (31.1%). The radiological appearances of the tumor were nodular (45%) and infiltrative type HCCs (55%). Concerning tumor extent, 44 cases (21.1%) were solitary large HCCs. In 97 cases (46.4%), the patient’s magnitude of tumor burden was calculated by the sum of the diameter of the largest tumor (in centimeter), and the number of tumors was > 7 (i.e., up-to-7 criteria).

**Table 1:** Characteristics of 209 patients.

Age (years), median (range)	74 (40–90)	Radiological appearance, n (%)	
Male, n (%)	158 (75.6)	Nodular type	94 (45.0)
BMI (kg/m^2^), mean ± SD	23.3 ± 0.3	Infiltrative type	115 (55.0)
Underlying liver disease, n (%)		AFP (ng/dl) (%)	
Alcohol	40 (19.1)	< 200	171 (81.8)
HBV	25 (12.2)	≧ 200	38 (18.2)
HCV	69 (33.0)	DCP (mAU/ml), n (%)	
NASH/NAFLD	41 (19.6)	< 400	142 (70.0)
PBC	3 (1.4)	≧ 400	61 (30.0)
Cryptogenic	31 (14.8)	Number of tumors, n (%)	
Child–Pugh class, n (%)		≤ 3	93 (44.5)
A	179 (85.6)	4–6	89 (42.6)
B	30 (14.3)	> 7	27 (12.9)
mALBI grade, n (%)		1 (solitary large)	44 (20.5)
1	93 (44.5)	Largest tumor diameter, n (%)	
2a	45 (21.5)	≤ 3	98 (46.9)
2b	69 (33.0)	3–6	71 (34.0)
3	2 (1.0)	> 6	40 (19.1)
Recurrence after curative therapy, n (%)	103 (49.3)	Up-to-7 criteria, n (%)	
Previous treatment, n (%)		Within	112 (53.6)
Surgical resection	26 (25.2)	Beyond	97 (46.4)
Ablation	42 (40.8)		
TACE	32 (31.1)		
SRT	3 (2.9)		

BMI, body mass index; HBV, hepatitis B virus; HCV, hepatitis C virus; NASH, nonalcoholic steatohepatitis; NAFLD, nonalcoholic fatty liver disease; PBC, primary biliary cholangitis; TACE, transarterial chemoembolization; SRT, stereotactic radiotherapy; AFP, alpha-fetoprotein; DCP, des-γ-carboxy prothrombin.

### Risk factors of treatment refractoriness

**Figure 2:**
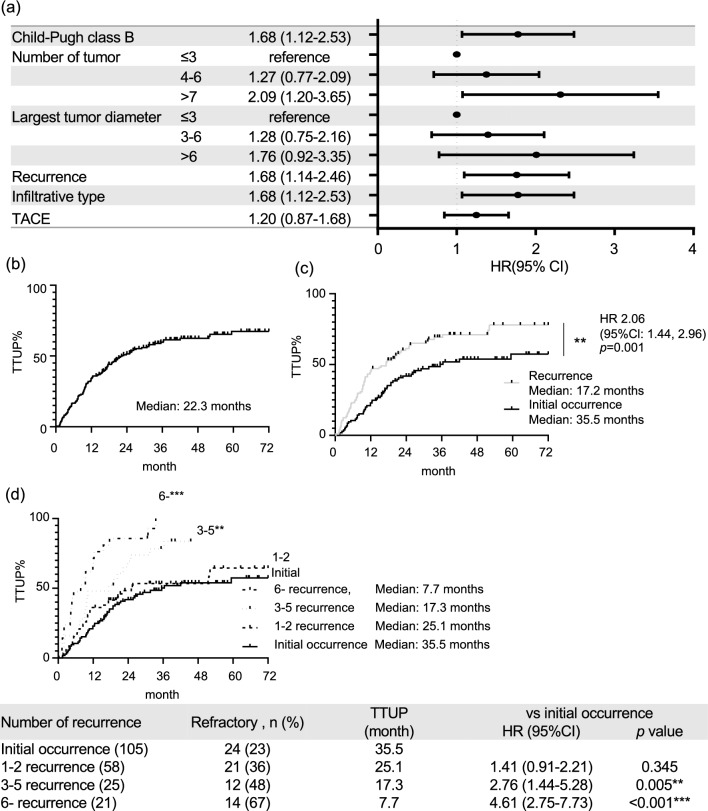
(**a**) Forest plot with hazard ratios based on multivariate Cox regression analysis about TTUP. Kaplan–Meier curves of the TTUP in (**b**) all patients, (**c**) patients who underwent locoregional and surgical therapies because of initial occurrence or consecutive recurrence, and (**d**) patients who underwent locoregional and surgical therapies for each number of recurrences after curative therapy. Asterisks indicate statistically significant differences of means (0.01 ≤ * *P* < 0.05; 0.001 ≤ ** *P* < 0.01; *** *P* < 0.001). TTUP: time to untreatable progression by TACE, vs: versus.

Of the 209 patients, 72 (34.4%) were classified as refractory and 137 (65.6%) as responders to locoregional and surgical therapy. No patient died prior to untreatable progression by TACE within one year. We analyzed the predictive factors associated with refractory disease from the baseline parameters at inclusion (Table 2). Univariate analysis revealed that the Child–Pugh score/class (P = 0.007), number of tumors (P < 0.001), infiltrative radiological type (P < 0.001), and recurrence (P < 0.001) were significant factors for predicting treatment refractoriness. Furthermore, these factors were analyzed using multiple regression analysis, which revealed that all factors were confirmed as independent factors for predicting treatment refractoriness. The OR were 1.85 for the Child–Pugh score/class (95% CI, 1.23–2.77; P = 0.002), 1.22 for the number of tumors (95% CI, 1.06–1.42; P =  < 0.001), 12.5 for the infiltrative radiological type (95% CI, 5.39–28.9; P < 0.001), and 2.55 for recurrence (95% CI, 1.15–5.68; P = 0.022). Internal validation was performed using 1,000 bootstrapped samples, as shown in Supplementary Table 1. In addition, these factors were also extracted by the site respective analyses except Child–Pugh score/class, as shown in Supplementary Table 2 and Fig. 2a. We focused on recurrence as a novel factor that predicts treatment refractoriness and performed the following analyses.

**Table 2:** Factors contributing to treatment refractoriness.

	Refractory	Responder	Univariate	Multivariate	
Variable	(N = 72)	(N = 137)	*P* value	OR (95%CI)	*P* value
Age (years), median (range)	74 (51–88)	74 (40–90)	0.937		
Male, n (%)	51 (70.8)	107 (67.7)	0.309		
BMI (kg/m^2^)	22.9 ± 0.8	23.0 ± 0.6	0.897		
Hepatitis B, C virus infection, n (%)	35 (48.1)	59 (43.1)	0.467		
Child–Pugh score	5.81 ± 0.10	5.46 ± 0.07	0.007**	1.85 (1.23–2.77)	0.002**
ALBI score	− 2.35 ± 0.06	− 0.248 ± 0.04	0.084		
Number of tumors	6.0 ± 0.4	3.3 ± 0.3	< 0.001***	1.22 (1.06–1.42)	< 0.001***
Largest tumor diameter	3.8 ± 0.3	4.4 ± 0.4	0.161		
Solitary large HCC	14 (19.4)	30 (14.4)	0.724		
Infiltrative type, n (%)	60 (83.3)	54 (39.4)	< 0.001***	12.5 (5.39–28.9)	< 0.001***
AFP (ng/dl)	662 ± 510	594 ± 370	0.914		
DCP (mAU/ml)	3752 ± 1828	4324 ± 1311	0.799		
Recurrence	48 (66.7)	56 (40.9)	< 0.001***	2.55 (1.15–5.68)	0.022*
TACE, n (%)	54 (75.0)	85 (62.0)	0.066		
Surgical resection, n (%)	11 (15.3)	35 (25.6)	0.114		
Ablation, n (%)	7 (9.7)	17 (12.4)	0.653		
Prior history of TACE, n (%)	14 (29.2)	18 (32.1)	0.832		

Asterisks indicate statistically significant differences of means (0.01 ≤ * *P* < 0.05; 0.001 ≤ ** *P* < 0.01; *** *P* < 0.001).

### Treatments outcomes of later times of recurrence

**Figure 3:**
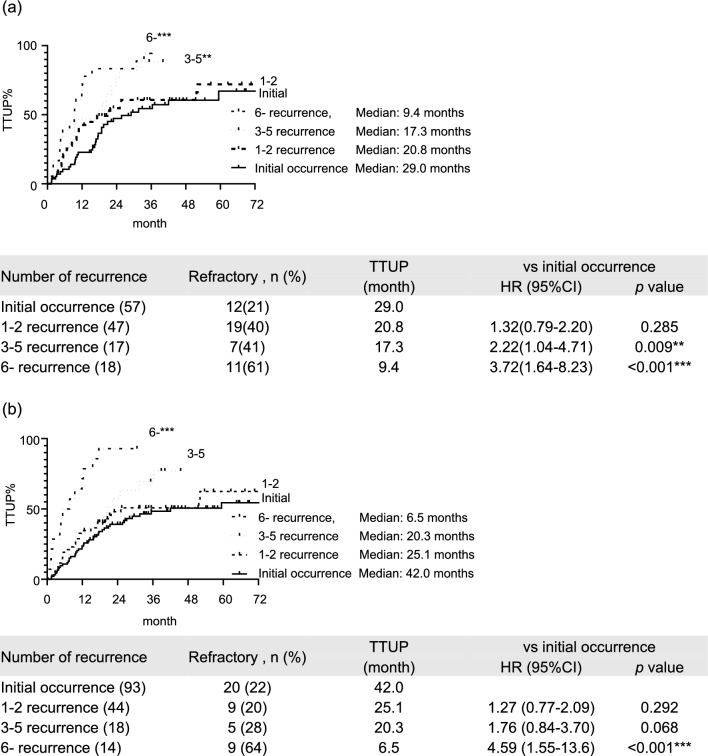
Kaplan–Meier curves of the TTUP in (**a**) patients who underwent TACE (**b**) patients with Child–Pugh class A for each number of recurrences after curative therapy. Asterisks indicate statistically significant differences of means (0.01 ≤ * *P* < 0.05; 0.001 ≤ ** *P* < 0.01; *** *P* < 0.001). TTUP: time to untreatable unTACEable progression, vs: versus.

The predictive factors associated with TTUP were determined from the parameters identified in Table 2 using Cox proportional hazards models (Fig. 2a). The HR were 1.68 for the Child–Pugh B (95% CI, 1.12–2.53), 2.09 for the number of tumors > 7 (95% CI, 1.20–3.65), 1.68 for the infiltrative radiological type (95% CI, 1.12–2.53), and 1.68 for recurrence (95% CI, 1.14–2.46). The TTUP was 22.3 months in the entire cohort (Fig. 2b). In the recurrence subgroup, the TTUP was 17.2 months and thus significantly inferior to the TTUP in the initial occurrence subgroup, which was 35.5 months (hazard ratio [HR], 2.06; 95% CI, 1.44–2.96; P = 0.001) (Fig. 2c). Furthermore, the TTUP was significantly inferior to the TTUP in the initial occurrence subgroup in the 3–5 recurrence (17.3 months; HR, 2.76; 95% CI, 1.44–5.28; P = 0.005) and > 6 recurrence subgroups (7.7 months; HR, 4.61; 95% CI, 2.75–7.73; P < 0.001) (Fig. 2d). The TTUP decreased in cases of later recurrence. The results were confirmed in each site by the respective analyses, as shown in Supplementary Fig. 1a–c. Subgroup analysis only of TACE, the TTUP was significantly inferior to the TTUP in the initial occurrence subgroup (29.0 months) in the 3–5 recurrence (17.3 months; HR, 2.22; 95% CI, 1.04–4.71; P = 0.009) and > 6 recurrence subgroups (9.4 months; HR, 3.72; 95% CI, 1.64–8.23; P < 0.001) (Fig. 3a). Subgroup analysis for Child A patients, the TTUP was significantly inferior to the TTUP in the initial occurrence subgroup (42.0 months) in the > 6 recurrence subgroups (6.5 months; HR, 4.59; 95% CI, 1.55–13.6; P < 0.001) (Fig. 3b).

### Patterns of later times of recurrence

**Figure 4:**
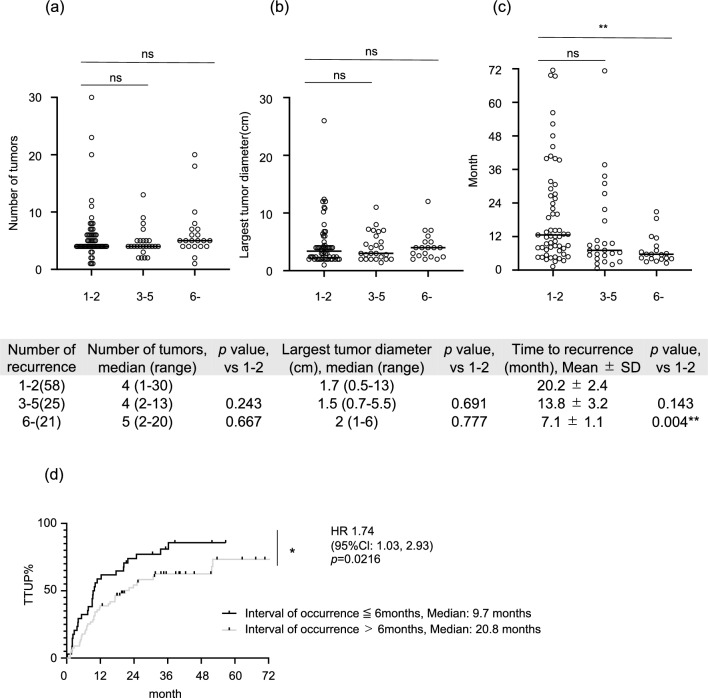
Patterns of recurrent tumor conditions for each number of recurrences after curative therapy. (**a**) Number of tumors. (**b**) Largest tumor diameter. (**c**) Time to recurrence. (**d**) TTUP in interval of occurrence ≦ 6 months vs > 6 months. Asterisks indicate statistically significant differences of means (0.01 ≤ * *P* < 0.05; 0.001 ≤ ** *P* < 0.01; *** *P* < 0.001).

We analyzed the tumor conditions of patients with recurrent HCC for each time of recurrence (three groups: 1–2, 3–5, and ≥ 6 recurrences). Although the number of tumors and the largest tumor diameter were comparable in each number of recurrences (Fig. 4a, b), the interval of occurrence from previous curative therapy tended to be shorter in later occurrent times, and patients with > 6 recurrences showed significantly shorter intervals of occurrence than those with 1–2 recurrences (Fig. 4c). Moreover, in cases where the interval of occurrence was less than 6 months, TTUP was significantly shorter compared to others (Fig. 4d).

### Treatment responsiveness of infiltrative HCC

**Figure 5:**
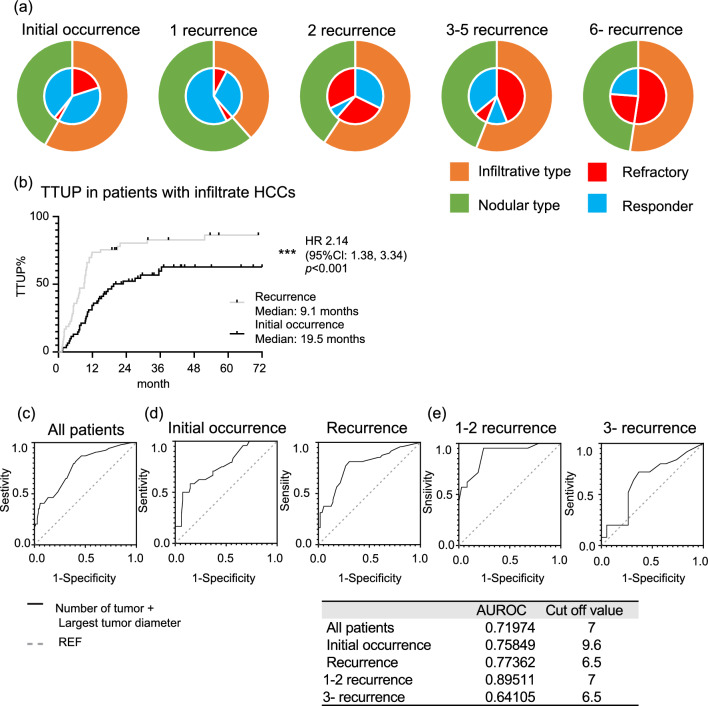
Differences in therapeutic response according to radiological features in each number of recurrences. (**a**) Proportion of therapeutic response according to radiological features in each number of recurrences. (**b**) Comparison of TTUP between initial occurrence and recurrence in patients with infiltrative HCCs. ROC analysis for predicting treatment refractoriness and comparison of cut-off values of the sum of the diameter of the largest tumor (in centimeter) and the number of tumors for predicting treatment refractoriness in (**c**) all patients, (**d**) initial occurrence vs recurrence, (**e**) 1–2 recurrence vs 3- recurrence. Asterisks indicate statistically significant differences of means (0.01 ≤ * *P* < 0.05; 0.001 ≤ ** *P* < 0.01; *** *P* < 0.001). TTUP: time to untreatable untraceable progression, HCCs: hepatocellular carcinomas. ROC: receiver operating characteristic. Asterisks indicate statistically significant differences of means (0.01 ≤ * *P* < 0.05; 0.001 ≤ ** *P* < 0.01; *** *P* < 0.001). TTUP: time to untreatable progression by TACE, HCCs: hepatocellular carcinomas.

Of 115 patients with infiltrative radiological features of HCC, 40 (65.6%) patients with initial occurrence, 8 (80%) patients with 1 recurrence, and 9 (47.4%) patients with 2 recurrences were classified as responders. However, in patients with later recurrence with infiltrative radiological features of HCC, only 3 (12%) patients with ≥ 3 recurrences were classified as responders (Fig. 5a). In the initial occurrence subgroup, the TTUP was 19.5 months and thus significantly superior to the TTUP in the recurrence subgroup, which was 9.1 months (HR, 2.14; 95% CI, 1.38–3.34; P < 0.001) (Fig. 5b). The TTUP was also significantly longer in patients with initial occurrent HCC than in those with recurrent HCC who even had an infiltrative type of HCC.

### Cut-off value of the magnitude of tumor burden in the recurrent condition

Regarding the magnitude of tumor burden, the number of tumors was identified as a risk factor for treatment refractoriness in this cohort. However, inclusion of the largest tumor diameter and prognostic values improved more than the number of tumors alone, as shown in Supplementary Fig. 2. Sensitivity (from 48.6 to 77.8%), negative predictive values (from 74.3 to 83.0%), and AUROC (from 0.64 to 0.72) were improved.

ROC analysis and the Youden index were used to determine the optimal cut-off values for predicting treatment refractoriness based on the sum of the diameter of the largest tumor (in centimeter) and the number of tumors. The cut-off values for predicting treatment refractoriness of tumor burden were 7 in all patients (Fig. 5c). In more detail, the cut-off values were 9.6 in the patients who had initially occurrent HCC and 6.5 in the patients who had recurrent HCC (Fig. 5d). Moreover, the cut-off values of tumor burden were 7 in the patients who had 1 or 2 recurrent episodes of HCC and 6.5 in the patients who had ≥ 3 recurrent episodes of HCC (Fig. 5e).

## Discussion

Treatment refractoriness in patients with HCC appears with early tumor progression or transient deterioration of liver function in Child–Pugh score/class C^33^. We investigated the Child–Pugh score/class, number of tumors, infiltrative radiological features, and recurrence as relevant factors associated with early recurrence and extreme progression after TACE, thermal ablation and surgical therapy in patients with BCLC intermediate-stage HCC. Notably, recurrence was identified as an independent factor from other factors such as the number of tumors or infiltrative radiological features, as shown in Table 2 and Fig. 2a. The median period to the TTUP was significantly shorter in patients with recurrent HCC than in those with initial HCC (Fig. 2c). Based on the result that prior TACE history was not identified as a relevant factor for predicting treatment refractoriness, even if radical treatment such as resection or ablation was achieved in previous treatment, patients with recurrent HCC were found to have a poor prognosis.

Moreover, our study also showed that the TTUP decreased in cases of more later times of recurrence (Fig. 2d). Patients with ≥ 3 recurrences showed a significantly shorter TTUP than patients with initial occurrent HCC. The subgroup analysis of patients who underwent TACE or those with Child A also showed similar results: TTUP decreased as the number of recurrences increased (Fig. 3a, b). As shown in Fig. 4a–c, although the number or size of the tumor was comparable in each number of recurrent times, the interval of occurrence from previous curative therapy tended to be shorter in later occurrent times. Patients with ≥ 6 recurrences showed a significantly shorter interval of occurrence than those with 1 and 2 recurrences of HCC. Infiltrative radiological features, which are considered poor prognostic factors, were also identified as relevant factors for treatment refractoriness in the current study. Although predicting the gross classification of HCC using only radiological imaging evaluation is challenging, studies have reported that macrovascular invasion can be predictable^34^. The results showed that radiological infiltration type contributes to TTUP, which may represent an important clinical relevance in predicting treatment refractoriness. The results shown in Fig. 5 are noteworthy; during the initial occurrence or early times or recurrence, first or second recurrence, these infiltrative types of HCCs responded to locoregional and surgical therapy to some extent. Similarly, the TTUP was also significantly longer in patients with initial occurrent HCCs than in those with recurrent HCCs who even had an infiltrative type of HCC. These results suggest that multiple asynchronous recurrences could serve as prognostic factors independent of radiological infiltrative types. The magnitude of tumor burden is the major predictive factor for treatment refractoriness. The number of tumors was also identified as a relevant factor for treatment refractoriness in the current study. To predict TACE refractoriness, some studies reported the usefulness of up-to-7 criteria in patients with BCLC intermediate-stage HCC^16–18^. We obtained similar results; the cut-off value of magnitudes of tumor burden, which could predict refractoriness to locoregional and surgical therapy, was 7.0 in the size + number of tumors. These findings supported the notion that the up-to-7 criteria for predicting TACE refractoriness were also useful for predicting refractoriness to locoregional and surgical therapy in patients with BCLC intermediate-stage HCC. Additionally, focusing on the difference in the cut-off value of tumor burden between initial occurrent HCCs and recurrent HCCs, the cut-off value was increased to 9.6, especially in patients who had initially occurrent HCCs. This result was corroborated by previous studies that reported the cut-off value of magnitude of tumor burden up to 11 for predicting TACE refractoriness in patients with BCLC intermediate-stage HCC^35,36^ since they only included patients with initially occurrent HCCs. Moreover, our data indicated that in patients with multiple occurrent HCCs, the cut-off value of magnitude of tumor burden tended to decrease compared with that at early times of recurrence. These results suggest that the threshold of tumor burden should be lowered in patients with multiple asynchronous recurrences for the clinical decision of treatment refractoriness to locoregional and surgical therapy.

Multifocal HCC arises synchronously and manifests as either different tumors or intrahepatic metastases of primary HCC^37,38^. HCC with multi-centric occurrence and HCC with intrahepatic metastasis had quite different characteristics and clinical course. The prognosis of repeated hepatectomy is determined by the pathological type of multicentric occurrence or intrahepatic metastasis^39^ and recurrent intrahepatic metastatic HCC is a poor prognostic factor^40,41^. Discrimination between these two types of HCC is clinically crucial. Recent technological advances in next-generation sequencing and bioinformatic approaches have enabled elucidation of the genomic profile of HCC by whole-genome sequencing. The diversity of HCC has been explored, and several mutated genes have been identified, such as *TP53*, *CTNNB1*, *TERT*, and *ARID2*^42–44^. Sequencing the genetic alterations of multifocal or asynchronous recurrent HCCs could accurately differentiate between multicentric occurrence and intrahepatic metastasis^45^. It has also been reported that a patient with multi-centric occurrent HCC has distinct mutation spectrum of tumor-distinct mutation profiles, but has rates of comparable nonsynonymous to synonymous substitution. In contrast, intrahepatic metastatic HCC have similar mutation spectrum with significantly higher ratios of nonsynonymous to synonymous substitution in the metastatic lesions than in the primary tumor^46^. This previous finding may indicate that evolutional positive selection favors the proliferation of metastatic subclones, and the tumor acquires to increase its malignant potential. Discrimination of multi-centric occurrence and intrahepatic metastasis diagnosis is clinically made using the duration of disease-free period, tumor location, vascular invasion of the primary tumor, and hemodynamics based on CT or MRI examinations^47^. In our study, HCC with multiple asynchronous occurrences showed a short recurrence interval and poor prognosis. These patterns, which were observed in later times of recurrent HCC, resemble the intrahepatic metastatic type of HCC. Another previous report comparing the genetic features of recurrent HCCs revealed that approximately half of recurrent HCC cases were derived from the clonal lineage of the initial tumor, and early recurrence was more likely to be second primary tumors that behave as progressive recurrence^48^. This evidence also supports the hypothesis that multiple asynchronous occurrences may lead to progression in tumor characteristics that resemble the sequence of intrahepatic metastasis.

In summary, our findings indicate that in patients with multiple recurrent HCC, a short interval of recurrence behaves similar to intrahepatic metastasis and leads to a worse prognostic course. Previous studies have shown that even in tumors refractory to only locoregional and surgical therapies, complete response can be achieved with the combination of systemic chemotherapy^49^. Therefore, patients with multiple asynchronous recurrences should be considered for multidisciplinary treatment. Further investigations are needed to elucidate whether multiple metachronous recurrent HCCs are also resistant to systemic chemotherapy other than locoregional and surgical therapy.

### Limitations

Our study has some limitations. This retrospective study had inherent bias. Our study requires an external validation cohort to obtain more convincing results. Finally, a histological examination or sequencing of genetic alterations of tumors was not performed in this study, and the inclusion of such information is planned for future studies.

## Conclusions

In conclusion, this study’s findings suggest that recurrent HCC, especially at later times of recurrence, leads to increased treatment refractoriness to locoregional and surgical therapy. This information can be applied in clinical decision making for the early introduction of multidisciplinary treatment for patients who diagnosed as BCLC intermediate-stage HCC after multiple recurrent episodes.

### Supplementary Information

Supplementary Information.

## Data Availability

Research data are not publicly available on legal or ethical grounds. Further inquiries can be directed to the corresponding author.

## Abbreviations

HCC: Hepatocellular carcinoma
BCLC: The Barcelona Clinic Liver Cancer prognosis and treatment strategy
TACE: Trans-arterial chemoembolization
CR: Complete response
mRECIST: Modified Response Evaluation Criteria in Solid Tumors
RFA: Radiofrequency ablation
MWA: Microwave ablation
ALBI: Albumin-bilirubin score
mALBI: Modified albumin-bilirubin grade
TTUP: Time to untreatable progression
OR: Odds ratios
95% CIs: 95% Confidence intervals
ROC: Receiver operating characteristic
AUROC: Area under the receiver operating characteristic
HR: Hazard ratio
