# Supplementary material for: Multiple asynchronous recurrence as a predictive factor for refractoriness against locoregional and surgical therapy in patients with intermediate-stage hepatocellular carcinoma
Source: Sci Rep. 2024 May 13;14:10896. doi: 10.1038/s41598-024-61611-4 (PMC11091100; doi:10.1038/s41598-024-61611-4)
Supplement: Supplementary file 1 — Supplementary Information. [file 41598_2024_61611_MOESM1_ESM.pdf]

Supplementary Table 1. Bootstrap estimates for the multivariable models

Variable	OR	95%CI		*p* value
		lower	upper	
Child-Pugh score	1.849	1.234	2.771	0.003**
Number of tumor	1.224	1.057	1.418	0.007**
Infiltrative type, n (%)	12.482	5.385	28.935	<0.001***
Recurrence	2.550	1.146	5.675	0.022*

Statistics from 1000 bootstrapped samples were determined. Multivariate analysis of factors contributing to treatment refractoriness was performed. Asterisks indicate statistically significant differences of means (0.01 ≤ * $p < 0.05$; 0.001 ≤ ** $p < 0.01$; *** $p < 0.001$).

Supplementary Table 2. Characteristics of Tokyo, Saitama & Chiba cohort

Cohort	Tokyo	Saitama & Chiba	*p* value
Age (years), median (range)	74 (40-90)	75 (55-85)	0.4488
Male, n (%)	111 (74.0)	47 (79.7)	0.3850
BMI (kg/m2), mean ± SD	23.1 ± 0.4	23.5 ± 0.6	0.5969
Underlying liver disease, n (%)			0.0326*
Alcohol	26 (17.3)	14 (17.0)	
HBV	23 (15.3)	2 (3.4)	
HCV	49 (32.7)	20 (33.9)	
NASH/NAFLD	30 (20.0)	11 (18.6)	
PBC	2 (1.3)	1 (1.7)	
Cryptogenic	20 (13.3)	11 (18.6)	
Child-Pugh class, n (%)			0.8555
A	128 (85.3)	51 (86.4)	
B	22 (14.7)	8 (13.6)	
mALBI grade, n (%)			0.7505
1	64 (42.7)	29 (49.2)	
2a	37 (24.7)	8 (15.6)	
2b	47 (31.3)	22 (37.3)	
3	2 (1.3)	0 (0)	
Recurrence after curative therapy, n (%)	79 (52.7)	24 (40.7)	0.1187
Previous treatment, n (%)			0.4253
Liver resection	17 (21.5)	9 (37.5)	
Ablation	35 (44.3)	7 (29.2)	
TACE	24 (30.4)	8 (33.3)	
SRT	3 (3.8)	0 (0)	
Radiological appearance, n (%)			0.1968
Nodular type	63 (42.0)	31 (52.5)	
Infiltrative type	87 (58.0)	28 (47.5)	
AFP (ng/dl) (%)			0.6858
<200	125 (83.3)	46 (78.0)	
≧200	25 (16.7)	13 (22.0)	
DCP (mAU/ml), n (%)			0.3080
<400	107 (71.3)	35 (66.0)	
≧400	43 (28.7)	18 (34.0)	
Number of tumor, n (%)			0.4590
≤3	66 (44.0)	27 (45.8)	
4-6	64 (42.6)	25 (42.4)	
>7	20 (13.3)	7 (11.9)	
1 (solitary large)	34 (22.7)	10 (16.9)	
Largest tumor diameter, n (%)			0.1298
≤3	74 (49.3)	24 (40.7)	
3-6	50 (33.3)	21 (33.6)	
>6	26 (17.3)	14 (23.7)	
Up-to-7 criteria, n (%)			0.6826
Within	86 (57.3)	26 (44.1)	
Beyond	64 (42.7)	33 (55.9)	

BMI: body mass index, HBV: hepatitis B virus, HCV: hepatitis C virus, NASH: nonalcoholic steatohepatitis, NAFLD: nonalcoholic fatty liver disease, PBC: primary biliary cholangitis, TACE: transarterial chemoembolization, SRT: stereotactic radiotherapy, AFP: alpha-fetoprotein, DCP: des-γ-carboxy prothrombin.

(a)

Variable	Tokyo cohort Refractory (N=55)	Tokyo cohort Responder (N=95)	Tokyo cohort Univariate *p* value	Saitama & Chiba cohort Refractory (N=17)	Saitama & Chiba cohort Responder (N=42)	Saitama & Chiba cohort Univariate *p* value
Child-Pugh score	5.85 ± 0.12	5.48 ± 0.09	0.019*	5.65 ± 0.19	5.40 ± 0.12	0.280
Number of tumor	6.3 ± 0.6	3.3 ± 0.4	<0.001***	5.1 ± 0.6	3.5 ± 0.4	0.023*
Infiltrative type, n (%)	47(85.5)	39 (41.1)	<0.001***	13(76.5)	15(35.7)	0.009**
Recurrence after curative therapy	37(67.3)	42(44.2)	0.007**	11(64.8)	14(33.3)	0.042*

(b)

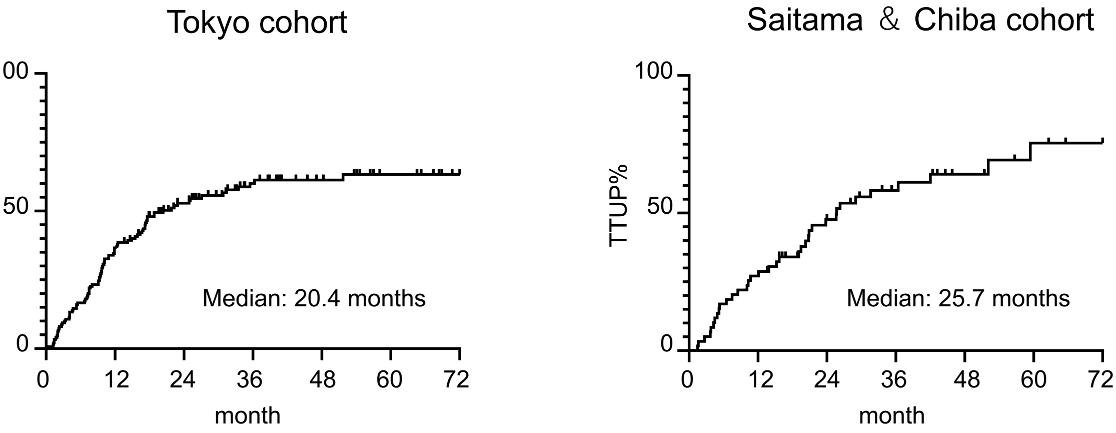

(c)

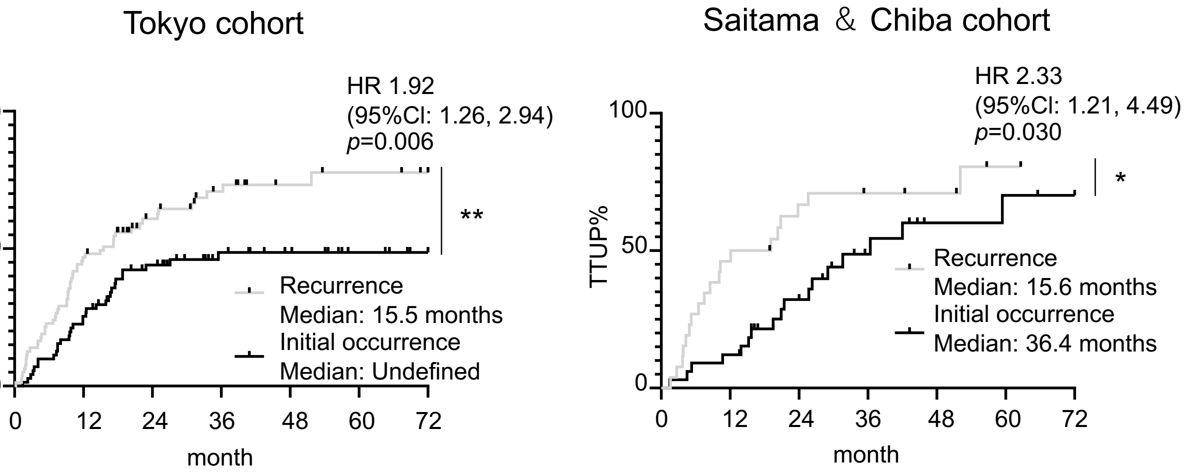

Supplementary Figure 1. Respective analysis by site of Tokyo cohort and Saitama & Chiba cohort (a) Univariate analysis of factor contributing to treatment refractoriness. Kaplan–Meier curves of the TTUP in (b) all patients, (c) patients who underwent locoregional therapies because of initial occurrence or consecutive recurrence. Asterisks indicate statistically significant differences of means (0.01 ≤ * p < 0.05; 0.001 ≤ ** p < 0.01; *** p < 0.001). TTUP: time to untreatable unTACEable progression.

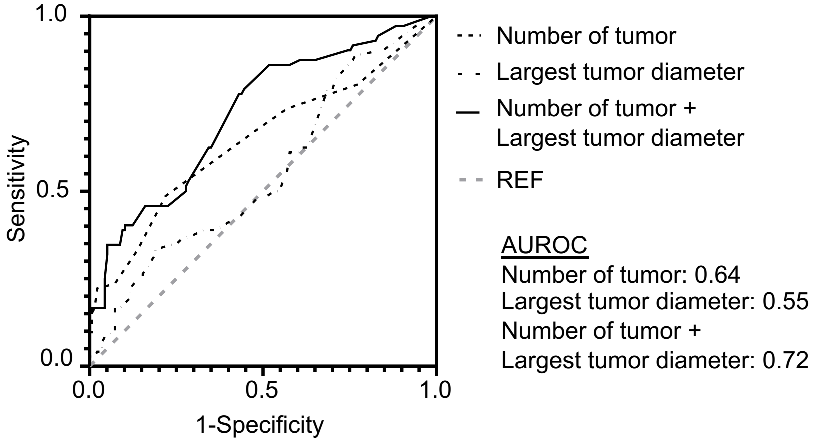

Supplementary Figure 2. ROC analysis for predicting treatment refractoriness
Comparison of number of tumors, largest tumor diameter, and sum of the diameter of the largest tumor (in centimeter) and the number of tumors for predicting treatment refractoriness calculated by ROC analysis.
ROC: receiver operating characteristic.
